# Surface Roughness and Grain Size Variation When 3D Printing Polyamide 11 Parts Using Selective Laser Sintering

**DOI:** 10.3390/polym15132967

**Published:** 2023-07-06

**Authors:** Riccardo Tonello, Knut Conradsen, David Bue Pedersen, Jeppe Revall Frisvad

**Affiliations:** 1 Department of Applied Mathematics and Computer Science, Technical University of Denmark, 2800 Kongens Lyngby, Denmark; rict@dtu.dk (R.T.); knco@dtu.dk (K.C.); 2 Department of Civil and Mechanical Engineering, Technical University of Denmark, 2800 Kongens Lyngby, Denmark; dbpe@dtu.dk

**Keywords:** additive manufacturing, SLS, PA11, surface roughness

## Abstract

Selective laser sintering (SLS) is a well-established technology that is used for additive manufacturing. Significant efforts have been made to improve SLS by optimizing the powder deposition, laser beam parameters, and temperature settings. The purpose is to ensure homogeneous sintering and prevent geometric and appearance inaccuracies in the manufactured objects. We evaluated the differences in the surface roughness and grain size of curved objects manufactured by using upcoming SLS technology that features two CO laser sources. Our analysis was carried out on polyamide 11 (PA11), which is a sustainable biobased polymer that has been gaining popularity due to its high-performance properties: its low melting point, high viscosity, and excellent mechanical properties. By using a Taguchi experimental design and analysis of variance (ANOVA), we examined the influence on the surface roughness and grain size of the build setup, the presence of thin walls, and the position of the sample on the powder bed. We found significant differences in some surface roughness and grain size measurements when these parameters were changed.

## 1. Introduction

While selective laser sintering (SLS) is used in additive manufacturing to produce parts with high surface quality, these parts have greater surface roughness and grain size than those produced by other polymeric additive manufacturing techniques [1]. Although a smooth surface is not always preferred by consumers, surface roughness and grain size are properties of 3D-printed parts that consumers notice during tactile and visual assessments [2,3]. The surface roughness of parts produced by SLS 3D printers results from the SLS process, which uses a high-power laser to sinter granular polymer powder into a solid structure. The surface texture quality of SLS-printed parts is affected by many parameters, including the preparation method, the equipment utilized (powder properties, machine setup and processing parameters), and the position and orientation of the part inside the build chamber [1,4,5]. In this study, we use laser confocal microscopy to find correlations between surface roughness and grain size measures at different levels of magnification and printing parameters that are related to the build setup, presence of thin walls in a part, and position on the build plate.

**Figure 1:**
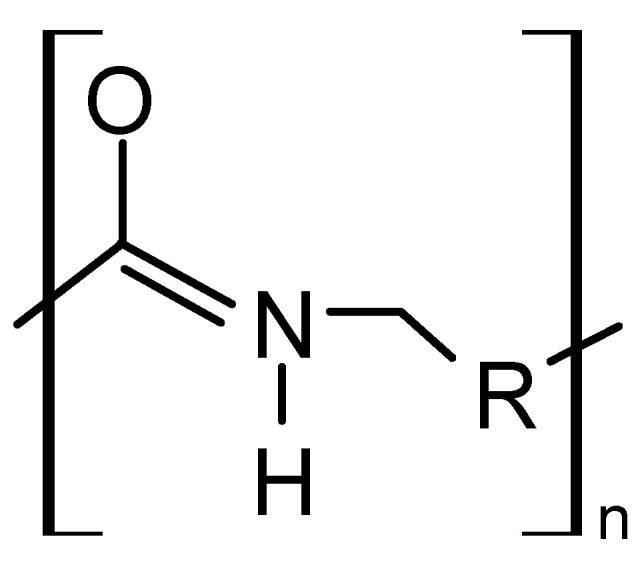
Chemical structure of PA.

Polyamide (PA) is the most commonly used material in SLS and is mainly used in the form PA12, PA11, and PA6 [6]. Polyamide is also known as nylon, which indicates a synthetic polyamide, and the two terms are often used interchangeably. In this study, we use PA11 powder or PA1101, which is a rebranded type of Rilsan Invent Natural (Arkema) from EOS [7]. While PA is mainly manufactured from petrochemical sources, ongoing research is creating drop-in materials for PA and completely new biobased materials [8]. Polyamide 11 (PA11) is a well-established biobased material [9] formed by the polycondensation of ω-aminocarboxylic acids to create a linear polymer chain with a characteristic recurring functional acid–amide group (Figure 1) with alkyl group chains (R groups) from the reactants [10]. Lately, the polycondensation of ω-aminocarboxylic acids has become more popular due to an increasing interest in biobased aliphatic polyamides. PA11 is synthesized by using 11-aminoundecanoic acid from castor oil as a monomer and multifunctional agents [8,11]. A PA11 powder is generated by either milling or grinding procedures, spray drying, or precipitation from solvents [12]. The result is a semicrystalline thermoplastic, making it suitable for SLS printing purposes [7,8].

To be processed, the semicrystalline powder requires higher temperatures than alternative materials, but it provides higher tensile properties [13]. The crystallization of PA11 starts with the cooling of the polymer chains. When synthesized, the chains appear to be disordered and amorphous, but as they cool down, they start arranging themselves into a repeating, ordered structure. The degree of crystallinity in PA11 can vary depending on several factors, including the cooling rate [14,15], molecular weight [16], and the presence of any additives [17]. The PA11 powder is also known for its good dimensional stability under fluctuating humidity and good mechanical properties, including its high strength, stiffness, and abrasion resistance [6,7].

The powder is important for the selective laser sintering process. The key factors of flowability and packing density during the SLS process, in particular the re-coating of the powder bed, are affected by the powder’s particle size distribution and shape (where more narrow and rounded particles are preferable) [12,18,19], surface roughness and interparticle forces, and moisture and temperature [19,20,21,22,23]. In SLS printing, after completing a part, a common procedure is to recycle the remaining unused powder. The reuse of aged polyamides is currently a significant area of research. In fact, to facilitate printing and ensure better dimensional stability, parts are printed slightly below the powder’s melting temperature, and this causes unsintered powder in the build chamber to undergo alterations in its thermal and mechanical properties, making its reuse challenging [24,25]. Thus, before reuse, the old powder needs to be sieved and mixed with new powder in appropriate ratios [24].

Printing in 3D with SLS is performed by using successive layers of powdered material delivered either by a blade from a hopper (powder reservoir) or by a roller from a powder feeder to the powder bed. The powder can be preheated to around 100 ${}^{\circ }$C in the hopper/feeder to achieve the best flowability and packing density by reducing the Hausner factor [26] below 1.25 and the granular Bond number below 100 in the unconsolidated material [23,27]. The granular Bond number is the ratio of the interparticle forces to the contribution of gravity in two particles. Moreover, preheating the feeder/hopper reduces the temperature difference between the feeding system and the powdered layer so that the surface of the powder bed can sinter, which decreases the undercooling effect in the molten layer and prevents the sintered layer from curling or warping [20,28,29,30,31].

Along with the preheating of the powder, the bed and the chamber are also preheated, but to a temperature right below the melting point (for PA11, the melting point is 201 ${}^{\circ }$C, but the temperature is kept at 180 ${}^{\circ }$C). This is performed to increase the energy efficiency of the printing process. The laser can then transform the material into its molten state by increasing the temperature by just a few degrees. This relaxes internal stresses in the part and prevents warping [31,32]. In an optimal sintering window, the hysteresis between melting and crystallization inhibits the crystallization and the solidification of the layers until all of them are sintered and the powder is kept in a melt state with low viscosity [22,30,31,33]. Schmidt et al. [18] showed that if the sintering temperature is too close to the crystallization peak, premature crystallization occurs and the printed part curls and becomes distorted. If the sintering temperature is instead too close to the melting temperature, a loss in the accuracy of the part features occurs. On average, if there is a large difference between the onset melting temperature and the onset crystallization temperature, the crystallization of the polymer melt is reduced. This reduced crystallization helps to decrease the shrinkage of the printed parts, hence reducing their internal stresses [34].

Keeping the part being printed at a constant temperature for the entire process is challenging. Strano et al. [35] found that the preheating energy is proportionally related to the number of sintered layers and the build orientation of the part. The build chamber plays an important role in the sintering of the part. Melt inhomogeneities are verifiable along the *z*-axis (up) due to the nature of the process [22,36] and in the xy plane (bed) [37,38]. The latter are probably due to the heating devices being unable to uniformly cover the entire planar surface with the same temperature distribution. The lack of melt homogeneity affects the part being printed; for example, inconsistencies in cooling and crystallization rates can cause warping effects in the lower area of the part [39]. In addition, an increase in the average molecular weight of the PA11 can positively shift the crystallization toward lower temperatures and the melting point towards higher temperatures, which hinders the particles in coalescing, hence disrupting the surface quality of the part [40].

Once the sintering process has created a layer of the part, the powder bed is lowered by a height that corresponds to the layer thickness and a new powdered layer is deposited. The unsintered powder (powder cake) remains in place and provides structural support, which means printing supports are not required and unconventional printing directions are permissible, whereas they are generally prohibited by other 3D printing systems. For each layer deposited, the printing parameters, such as the bed temperature and removal chamber temperature, can vary. The removal chamber is physically separated from the powder by a steel plate. This is important for the cooling process. In fact, the removal chamber can normally be kept at a fixed temperature so the layer and its powder cake can cool down homogeneously without curling while the subsequent layer is processed [41]. However, there are different ideas on how to adjust this temperature to increase the efficiency and improve the cooling conditions for the part [42].

Once the part has been printed by stacking layers upon layers and it has a temperature theoretically equal to the removal chamber temperature, it is left to cool down homogeneously surrounded by its powder cake until an extraction temperature has been reached. When the piece is removed, the excess powder is moved to a recycling container for reuse. The cooling phase is another critical step in the SLS printing process, where crystallization is the main phenomenon; we refer to Amado [31] for more insights on this matter.

### 1.1. Sintering Process

**Figure 2:**
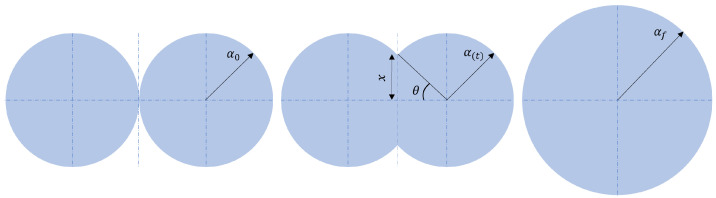
Schematic representation of the coalescence process. A droplet of radius $\alpha_{0}$ coalesces with another droplet and ends with the radius $\alpha_{f}$. During the process, the particle radius α and the angle of the intersection θ and the radius of the neck *x* change as a function of time *t*.

The main step in the SLS system is the sintering process [10,36,37,43]. The packing density and the viscosity in the molten state at the processing temperature are the most important parameters that define the sintered density [22,31]. Variations in the processing temperature change the viscosity and thus the rate of densification. The ratio between the viscosity of the melt and the surface tension has a big impact on the coalescence of the polymer powder particles. A higher ratio reduces the coalescence rate of particles during the sintering time, while a lower ratio stimulates the formation of droplets that thwart the homogeneity of the layer [31]. The coalescence of polymer powder particles is the main constituent of the sintering process. When the polymer powder particles are above their glass transition or melting temperature, they form necks to decrease their total surface area (Figure 2).

Frenkel [44] was the first to describe the neck formation phenomenon, Pokluda et al. [45] modified Frenkel’s findings by considering the variation in the radius of the particles over the whole SLS process, while Bellehumeur et al. [46] and later Scribben et al. [47] incorporated the viscoelastic nature of the molten polymers to obtain a more precise description of the process. The neck formation is activated by the energy density in the particles caused by the incident light, which is parameterized by the laser power and speed. The energy density leads to good or poor bonding between the layers. If the energy density is too low, delamination can occur between the layers, while if it is too high (low-speed, high-power laser) warping and curling are more frequent. In the case of non-adjusted combinations, balling is also possible (high-speed, high-power laser) [33,48]. Moreover, the energy density also affects the microstructure of the sintered part. In the case of a higher energy density, the cooling rate and crystallinity degree might cause the part to shrink more, resulting in a lower porosity as the pores become smaller or are absorbed [48]. This was confirmed by Kozior [5], who showed that a reduction in the energy density leads to higher stress relaxation and overall improved quality of the surface texture. However, Wang et al. [49] found that higher laser power leads to lower shrinkage of the part. This is due to the increased sintering width and depth caused by a higher power, which creates a higher sintered area, thus decreasing the heat exchange between the sintered area and the rest of the sintered layer.

**Figure 3:**
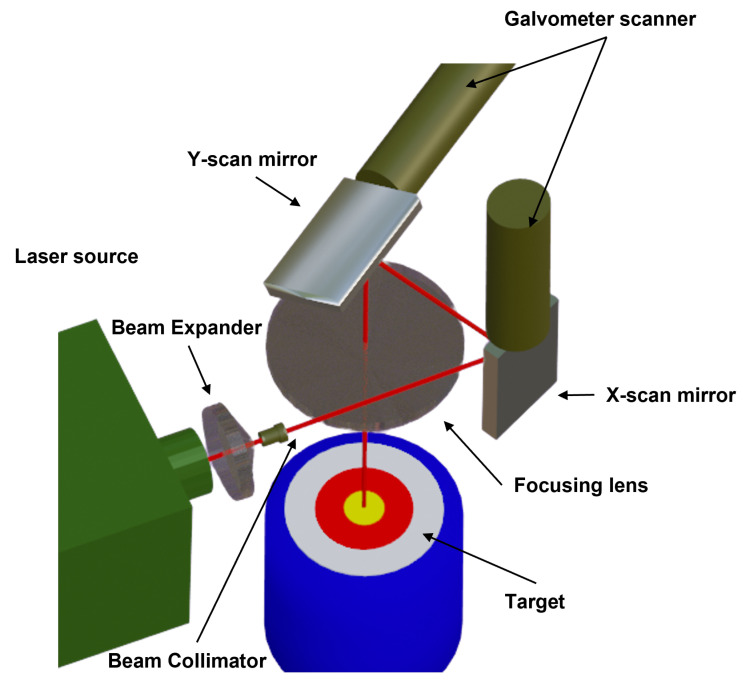
Schematic representation of the SLS technology.

The laser beam is steered to the point of the powder bed to be sintered. This is performed by a galvanometer scanning technology that is coupled with a beam expander and beam collimator to control the energy density, the diameter and the spot size of the laser beam, see Figure 3. As a laser source, CO${}_{2}$ and Nd:YAG lasers are mainly used, but CO${}_{2}$ is usually preferred as it performs better in SLS systems. However, EOS has recently designed the CO laser beam that we used, which seems to have a smaller light spot, higher processing efficiency, and larger processing range. The laser used irradiates the powder with a radial Gaussian distribution. Xin et al. [50] introduced a ray-tracing model that considers the attenuation of the laser energy in the powder bed, which can be described as a series of scattering effects. In this way, under the assumption of spherical particles, they found a strong dependency of the scattering on the distribution of the laser energy, resulting in variations of the temperature in different grains. Thus, as found experimentally also [51], there is a correlation between the angle of incidence and the laser irradiance and thus the sintering performance.

### 1.2. Related Work

As we have seen, laser power has an important influence on the outcome of the part. The relationship of laser power with the energy density creates a correlation with the laser scan speed. On average, slow scan speeds favour higher and more homogenous melting areas, which generate lower surface roughness [33]. However, the design of the part also has a big influence on the printing process: in the research that we discuss in the following, layer thickness, printing direction, and hatch spacing have been found to have a big impact on the shrinkage of the part and therefore its accuracy. In an analysis of variance (ANOVA), Sharma and Singh found that layer thickness had a more accurate result at around 0.1 mm [52], even though there was a decrease in the shrinkage rate for parts with thicker layers [49]. In addition, they found variations in the printing directions with a difference in accuracy between the *x*- and *y*-axes, as also noted by Senthilkumaran et al. [53] and Woerz and Drummer [54]. Hatch spacing is another important parameter. Its calibration can reduce shrinkage and porosity and the use of additional techniques, such as scanning mirror inertia compensation or skywriting [53], can enhance the effect. Contouring, instead of improving the accuracy, gives more noise on mean deviations per unit length worsening its overall value [53]. However, double scan on the same layer has been shown to improve it [55].

Based on the above considerations and research findings, we focused our research on extending the accuracy optimization to other design features to enhance the appearance of the parts. We consider the position of the part in the powder bed, the build setup and the presence of thin walls in the part. In existing work, the position of the part has been studied from the laser beam properties perspective [50,51], and from a mechanical point of view, with some interesting results on differences in the crystallization rate [56]. Concerning the build setup, studies have focused on the build orientation as an overall property for improving the mechanical response to forces and loads [57,58]. The approach of the present study resembles the works of Strano et al. [35] and Bacchewar et al. [59]. Strano et al. [35] proposed a model from different datasets of downward- and upward-oriented surfaces and experimental roughness measurements, and did so to describe the stair-stepping effect over inclination angles. Bacchewar et al. [59] defined a model, using an ANOVA analysis, that takes into account downward and upward setups and other parameters, including layer thickness, build orientation and laser power. Based on the two aforementioned studies, we also took into account the downward and upward setups, but we connected them to different design parameters, such as build position and presence of thin walls in the printed part and used a Taguchi method to study correlations between them. We did this to find relations between the roughness and design parameters. The aim is to establish information that can help SLS-printer users in ensuring they print parts at the desired surface smoothness or roughness.

## 2. Materials and Methods

We obtained test samples using an industrial-scale SLS 3D printer EOS P 500 Fine Detail Resolution (EOS GmbH, Krailling, Germany). According to the manufacturer, this printer combines *F*-theta lenses, which offer high performance in laser scanning, with two 50 watt power and 5 μm beam diameter CO lasers that can reach a theoretical laser spot size of 25 μm with a scan speed of 10 m/s (for the sake of completeness: the spot size is 2Fλ/D, where *F* is the focal length of the lens, λ is the wavelength of the laser, and *D* is the diameter of the laser beam). The printer produces parts with a minimum layer thicknesses of 40–60 μm and minimum wall thicknesses of 0.22 mm.

**Figure 4:**
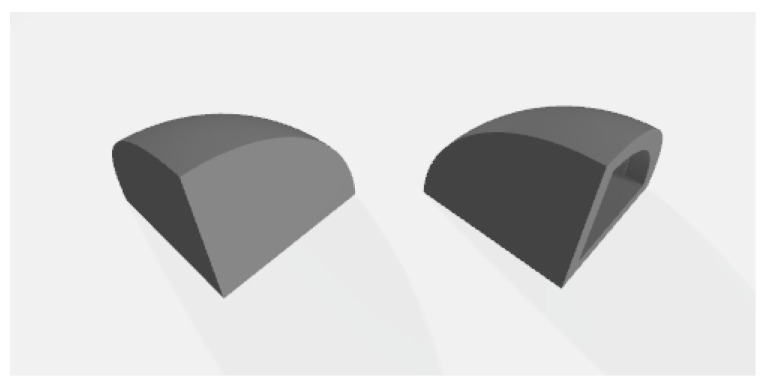
3D model of the samples. Full sample (**left**), hollowed sample (**right**).

The material used for printing was PA11 (PA1101) white. It has a glass temperature of 46 ${}^{\circ }$C and a melting temperature of 201 ${}^{\circ }$C. However, after 180 ${}^{\circ }$C the material starts absorbing energy endothermically (enthalpy of fusion) and the deflection pressure is minimal at 0.45 MPa. For this reason, the chamber and the powder bed are heated to 176 ${}^{\circ }$C and 175 ${}^{\circ }$C, respectively, in order to increase flowability, favour the first layer to stick to the powder bed and reduce stresses, including warpage. We note that, according to Tey et al. [34], PA11 has bimodal endothermic peaks for SLS systems, with the melting peak for the sintered part being the initial peak. This is due to larger unmelted powder particle cores that remained in the printed part due to insufficient heating. The removal chamber is kept at 160 ${}^{\circ }$C. Since we focused on the optimization of the part from an appearance perspective, two samples were designed with two different build setups for a quarter of a hemisphere with a radius of 20 mm: one solid (i.e., full) quarter hemisphere (Figure 4, left), and one hollowed on one side, creating thin walls of 2.5 mm (Figure 4, right). We selected thin walls of 2.5 mm as oversintering was observed locally for thinner features resulting in unsatisfactory surface quality. We wanted to test whether this phenomenon is partially still present with walls of 2.5 mm.

**Figure 5:**
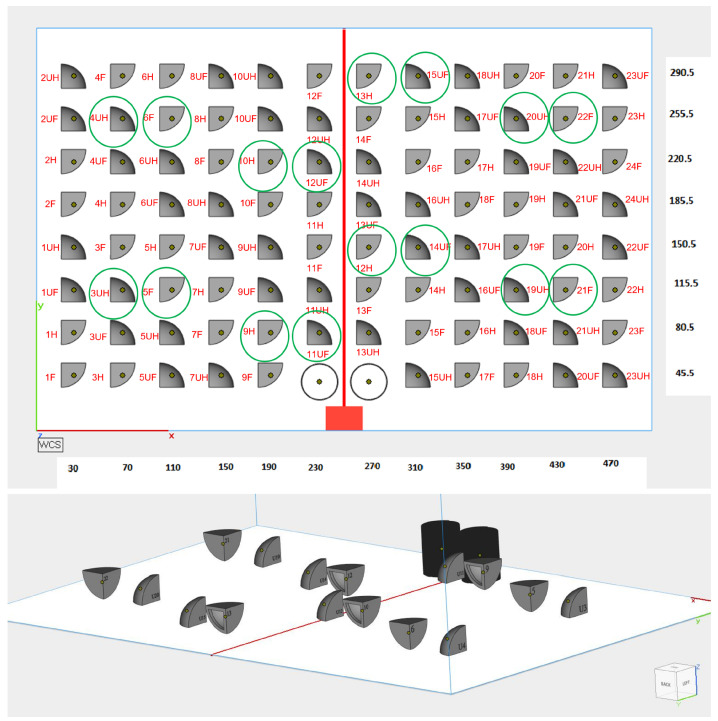
Layout of the print job for 94 samples on the same *z*-axis (**top**). The encircled samples were selected for analysis using confocal microscopy. Visualization in 3D of the 16 selected samples on the powder bed (**bottom**).

The samples were printed with the top edge of the part (Figure 4) towards the bed (direct-build setup) or pointing to the top of the machine (inverse-build setup). The quarter hemisphere part has three identical edges; therefore, in order to distinguish between them, we marked them with alphanumeric characters and oriented them on the left side of the machine (Figure 4). They were marked with an F for full, an H for hollowed and a U for inverse build setup (i.e., upside down). The printable volume measured 330 × 500 × 400 mm, so we requested a print job with 94 samples as in Figure 5.

The parts were printed with the top and bottom layers (commercially called upskin and downskin, respectively) at higher energy, to keep the part together and make the surface smoother. Contouring was not used, to avoid some particles being stuck at the border of the melting zone during the offset hatching of the laser, which would likely worsen the surface quality of the part. Instead, a double pass at a lower laser intensity was used for the contour to flatten all the unwanted stuck particles and smoothen the surface. Other specific printing parameters are protected by intellectual property and therefore not possible for us to share them. The parts were printed with commercial parameters, which means we used the best combination of parameters known by the manufacturer of the SLS 3D printer.

We recorded confocal microscope images with depth information using an LEXT OLS4100 (Olympus, Hamburg, Germany). Different sides of samples were imaged (top, curved side, two sides and bottom), with different levels of magnification (5×, 10×, 20×) and in different parts of the powder bed. SPIP 6.7.9 (Image Metrology, Kongens Lyngby, Denmark) and MountainsSpectral (Digital Surf, Besançon, France) were used to perform profilometry and particle analysis of the acquired images. When observing the curved surface (top), we selected a patch of observation as close as possible to the topmost pole of the sphere. This top view is very important for these samples because it reveals differences between the direct-build setup and the inverse-build setup. Moreover, the top view reveals any layer artefacts that may be present on the sample surface.

A Taguchi array design L16 (8^^^1 2^^^2) with three factors and 16 runs was applied to better understand the correlation between the factors. As indicated previously, the factors were the build position (with eight positions), the build setup (two, direct or inverse) and the presence of thin walls (two, full or hollowed). We investigated the roughness of the samples through the average roughness (Ra), the root mean square roughness (Rq), the arithmetical mean height (Sa) and the root mean square surface height (Sq) for the non-contact profilometer, repeating the measurements three times. Thus, the images were first levelled with the LS plane (least-squares plane) to find the primary surface and remove waviness, according to ISO 25178 [60]. After this, 3D images were generated and the correlated Sa and Sq were found. At this point, following the ISO 21920 [61] for roughness profiles, two filters were applied:

- Gaussian S-filter with cut-off wavelength at 2.5 μm from ISO 16610-21 [62] to remove the micro-roughness due to the instrument noise. We neglected this filter for the Sa, Sq analysis, as the micro-roughness due to the noise of the instrument was more dispersed and might cut out some imperfections of the samples.
- Gaussian L-filter with cut-off wavelength at 0.8 mm from ISO 16610-21 [62] to separate waviness from roughness. While the value does not accord with the specifications for mechanical and industrial components, our intent was to study the variance of the values among different samples and we thus preferred cut-off wavelengths that could better display the frequency of the roughness profile. Since 0.8 mm is a standard value, we applied it to curved and flat walls without distinction. It was not useful to apply this filter for the areal roughness, as we levelled the samples with an LS plane that could flatten a quarter of a hemisphere removing all the waviness.

We also applied the feature *manage end-effects* to cut out a portion of the filtered profile so that it was possible to visualize the full length without incurring distortion at the edges. Furthermore, a particle size analysis was carried out through the identification of D10, D50, D90, and Span for cumulative distribution and broadness with a watershed detection method for packed features. Size classification was only related to particles and the smallest boundary width was four pixels, where every pixel measures a 2.5 μm × 2.5 μm section. Moreover, shallow features were merged with a 5% range threshold. We acquired images from the top, the side where the mark was present and the bottom of the printed part.

As a first step after acquiring images, we used MountainsSpectral to define a template valid for all the samples with a profile both for curved and flat sides. In the study, the profile line traced for Ra, Rq measurements were taken diagonally for the curved sides, as the samples were analysed from the top. Differently, the profile line was taken with double orientation (vertical and horizontal line) for the flat and bottom sides. Table 1 lists most of our roughness and grain size observations. More data and images are available at a supplementary webpage (https://eco3d.compute.dtu.dk/sls-roughness/, accessed on 6 June 2023).

For the particle size analysis, we used SPIP 6.7.9 to obtain data valid for defining D10, D50, D90 (10% of the particles have diameters smaller than D10, 50% are smaller than D50, and 90% are smaller than D90), and Span, which is easily computed by

(1) $$\mathrm{Span}=\frac{D90-D10}{D50}\;.$$

The processed images from SPIP were analysed using OriginPro 2023 (OriginLab, Northampton, MA, USA).

The roughness values we measure are similar to or slightly smaller than those measured in other work on SLS [1]. Using a confocal laser scanning microscope on PA12-printed parts, Beitz et al. [63] found Ra roughness values in the range 24–31 μm, while Launhardt et al. [4] found Sa between 23 μm and 27 μm. For PA11, Ellis et al. [64] found Ra in the range 25–50 μm for the upskin (top side) and in 7–35 μm for the downskin (bottom side). Ours are in the range 8–23 μm for Ra and 9–35 μm for Sa.

**Figure 6:**
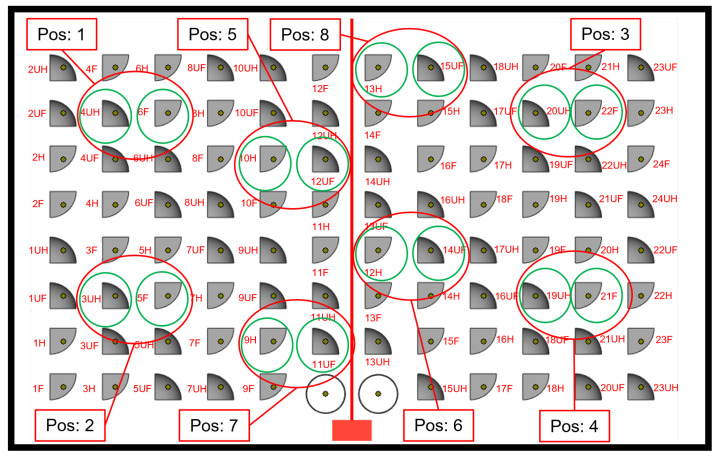
Numbers assigned to different positions in the powder bed for the *pos* factor in our factorial design. The red letter codes for the parts reveal their values for the build setup factor *bus* (U or not) and the design factor *fuh* (F or H).

To establish connections between the parameters and the surface analysis, we focus on a factorial design taking into account three factors: *pos* (position) at eight levels, *bus* (build setup, U or not) at two levels and *fuh* (part is full F or hollowed H), see Figure 6. A full factorial design, where we have measured the dependent variables at all combinations would require at least 8×2×2=32 trials, and if we want to estimate the means at all combinations this would leave no degree of freedom for estimating the error. In our case, we followed the Taguchi experimental design, having orthogonal arrays to organize the parameters that affect the process and the levels at which they vary. Therefore, we focused on pairs of combinations, with only 16 observations. To proceed with an orthogonal array, we had to assume that all or some interactions are zero. Afterwards, we analysed the arrays by performing an analysis of variance (ANOVA) for all 36 parameters (variables) measured using Statistical Analysis Software (SAS/STAT by SAS Institute).

As a procedure, we applied the general linear model (GLM), where the calculations were performed using the least squares regression approach. As a dependent variable, we chose any of the 36 parameters measured, while the three factors build position (with eight positions), the build setup (two, direct or inverse) and the presence of thin walls (two, full or hollowed) were considered independent variables. There is no natural ordering among the eight, two, and two levels, and thus they represent variation at a nominal scale. We denoted the observations $Y_{s,i},...,Y_{s,i}$ for *s* = 1, …, 16 as the number of samples and *i* = 1, …, 36 the parameter considered (Ra, Rq, Sa, Sq for top, side, and bottom at 5× magnification as well as 20× in the top view, D10, D50, D90, Span for top, side, and bottom at 20× magnification as well as 10× in the top view, both horizontal and vertical Ra and Rq for side and bottom). The expression for our model is:

(2) $$Y_{s}=\mu +{\mathrm{pos}}_{j}+{\mathrm{bus}}_{k}+{\mathrm{fuh}}_{l}+\epsilon_{s}$$

where μ represents the general level or intercept, ${\mathrm{pos}}_{j}$, ${\mathrm{bus}}_{k}$ and ${\mathrm{fuh}}_{l}$ are the parameter estimates or the contributions from each of the factors with *j* = 1, …, 8, *k* = 1, 2 and *l* = 1, 2 denoting the factor levels and $\epsilon_{s}$ are the random error component that are independent and Gaussian distributed with expectation 0 and variance $\sigma^{2}$ and *s* is the observation number.

## 3. Results

Each of the 16 analysed samples are described by three factors (independent variables determined before the experiments), and by 36 dependent variables measured during the experiment. The dependent variables are strongly correlated and we transform those into eight uncorrelated, latent variables called principal factors. They describe as much as possible of the total variation among the 36 dependent variables. The correlations between the principal factors and the original, dependent variables are the factor loadings. They are estimated with a principal factor solution and a VARIMAX rotation. The results show that 94.28% of the total variation is described by the first eight eigenvectors and we have four principal factors mainly related to Ra, Rq, Sa and Sq (surface roughness) and four principal factors related to D10, D50, D90 and Span (particle size distribution). The first principal factor (Factor1) is correlated with all the roughness measures of the bottom surface observed at 5× magnification and explains 18% of the variance. Similarly, Factor2 is correlated with all the roughness measures of the side surface at 5× magnification and explains another 18% of the variance. Factor3 is correlated with all the roughness measures of the curved top surface observed at 20× magnification as well as the Sa and Sq at 5× magnification. This factor explains 14% of the variance. Thus, a lot of the variance is due to surface roughness.

Calculating the strength of the relationship between the independent variables (position, build setup, and thin walls) and the observed variables or the principal factors, we find R-square values and *p* values for assessing the statistical significance of the relations, see Table 2. Although the first three principal factors explain half the variance, the differences in the means of these principal factors with respect to the independent variables are not statistically significant. For this reason, we focus on Factor4 and Factor7, which have relations with greater statistical significance than the other principal factors, and on those of the original dependent variables that exhibit statistically significant relations.

In the rotated factor pattern, see Table 3, Factor4 is correlated with the values of D10, D50, and D90 for a top view with 10× magnification (Top×10) and Factor7 is correlated with D10, D50, and D90 for a top view with 20× magnification (Top×20). This means that the overall size of grains observed on the surface at a medium scale (10× magnification) increases with increasing Factor4, while the overall size of grains observed at a finer scale (20× magnification) increases with increasing Factor7. We interpret a larger grain size as more coalescence (Figure 2), leading to larger particles. Surface roughness, on the other hand, is related to height variation in the surface and thus to the radius of the neck *x*. If *x* is small for many particles, roughness increases—even more for larger particles. In addition to Factor4 and Factor7, eight of the original dependent variables have significant relations to the input parameters (see Table 2). Four of those are described by Factor4 and Factor7, the remaining are Rq and Sq for Top×20, Rq for a bottom view with 5× magnification (Bottom×5) and Span for a bottom view with 20× magnification (Bottom×20).

**Figure 7:**
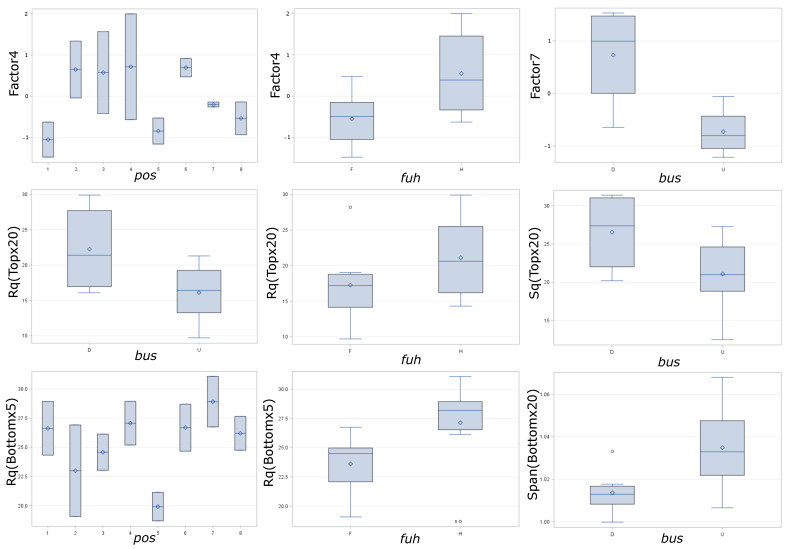
Boxplots for some of the variables exhibiting significant differences in means with respect to different explanatory variables (*pos*, *bus*, *fuh*).

With ANOVA we can say that our results are statistically significant, but we do not know exactly where the differences lie. For this reason, we apply a multiple comparison test, in particular Tukey’s HSD (honest significant difference), to find out which specific groups (compared with each other) are different. With Tukey’s HSD, we compare the largest sample mean with the smallest one. If we find a significant difference between the means, the test proceeds to greater sample means until the difference is non-significant. The main results of our multiple comparison test are listed in Table 4. By inspecting the boxplots of the variables with significantly different means for the different model input parameters (explanatory variables: *pos*, *bus*, *fuh*), the nature of the difference in the means becomes clear in some cases. Boxplots with a clear interpretation are shown in Figure 7.

## 4. Discussion

The multiple comparison test (Table 4 and Figure 7) allows us to draw some conclusions. For the curved surface (top view), we find that at the fine scale (Top×20) grain size (D10, D50, D90 represented by Factor7) and roughness (Rq, Sq) are most often larger for build setup D (tip toward the powder bed) than for U (tip towards the top of the machine). This indicates that, when inspecting fine details, we have too much coalescence and droplet formation with build setup D when compared with build setup U. We also find that the grain size observed at medium scale (Top×10) is likely smaller at position 1 as compared with positions 4 and 6 (see positions in Figure 6). This indicates that the laser sintering on the left-hand side results in less coalescence when operating at a longer distance, but this does not lead to a significantly different roughness. The coarse scale roughness of the bottom surface (Rq, Bottom×5), however, is most often lower in position 5 compared with position 7. Finally, the medium-scale grain size (Factor4) and the fine-scale roughness (Rq, Top×20) of the top surface, as well as the coarse-scale roughness (Rq, Bottom×5), are all often larger for hollow parts. The increase in the latter variable for hollow parts is particularly clear.

The thin walls in hollow parts have a significant influence on the final print. Tukey’s procedure reveals how D10, D50 and D90 of the top view with 10× magnification (Top×10) are consistently larger for hollowed parts. Conversely, the span of grain sizes at the fine level (Span, Bottom×20) is consistently larger for the inverse build setup (tip toward the top of the machine), while fine-scale particle sizes and roughness are smaller. However, further investigations need to be conducted to understand which process parameters can be changed to achieve more similar textures for these different configurations.

Some variables in the table are different for different powder bed positions. Overall, it seems that there are differences between the upper and lower parts of the powder bed and/or the left and right sides of the powder bed. This is supported by Factor4 and D10 of the top view with 10× magnification as well as the span of the bottom view with 20× magnification. However, we cannot conclude that the differences are specifically due to the upper/lower or left/right parts of the powder bed. Other parameters influence the result as well. The differences we observe might be due to different temperature gradients of the powder bed and the angle of incidence of the laser beam. Further investigations need to be conducted in order to understand whether, with modulation of the laser beam, the outcome might improve or not.

Profilometry assesses only a very small part of the surface area. Frequency analysis of the photos of the samples (seen in Table 1) might offer an alternative way of assessing the surface roughness and grain size distribution of the full sample, including points not directly analysed by profilometry. The power spectra of these images are related to frequency and amplitudes of the noise/grains in the image. We leave this type of analysis for future work.

## Figures and Tables

**Table 1:** Overview of our measurements. For each sample, we show two images of the curved top surface: a photo of the sample and a topography image of 2.5×2.5 mm${}^{2}$ (5× magnification). The orange colour scale in the topography image ranges from 0 μm (black) to 700 μm (white). We list the obtained roughness and grain size data for different sample views. Btm is short for Bottom. For Ra and Rq of the side and bottom surfaces we show an average of the two directions of observation.

Sample	View	Ra	Rq	Sa	Sq	D10	D50	D90	Span
3UH	Top×5	19.078	25.075	27.853	36.508				
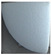	Top×10					9.029	13.669	20.819	0.8625
	Top×20	11.707	15.063	16.179	20.142	3.549	5.545	8.752	0.9383
	Side×5	17.334	23.487	20.053	27.045				
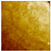	Side×20					3.476	5.545	9.093	1.013
	Btm×5	19.542	25.869	22.273	29.880				
	Btm×20					3.477	5.590	9.337	1.048
4UH	Top×5	21.341	28.023	29.216	37.889				
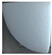	Top×10					8.573	13.078	19.994	0.8733
	Top×20	15.283	21.290	20.249	27.257	3.547	5.547	9.061	0.9941
	Side×5	19.512	24.865	22.071	28.542				
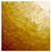	Side×20					3.622	5.854	9.554	1.013
	Btm×5	20.044	28.830	21.501	28.968				
	Btm×20					3.478	5.546	9.284	1.047
5F	Top×5	20.818	26.705	36.964	49.932				
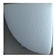	Top×10					8.691	13.156	19.697	0.8366
	Top×20	14.902	19.035	24.664	29.581	3.544	5.632	9.173	0.9995
	Side×5	20.144	25.708	21.937	28.165				
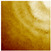	Side×20					3.758	6.024	10.017	1.039
	Btm×5	14.961	19.901	16.566	22.292				
	Btm×20					3.478	5.545	9.121	1.018
6F	Top×5	19.327	26.499	28.617	37.351				
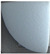	Top×10					8.461	12.926	19.592	0.8612
	Top×20	21.823	28.190	25.443	30.751	3.475	5.451	9.008	1.015
	Side×5	17.779	25.098	19.367	26.588				
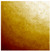	Side×20					3.404	5.409	8.782	0.994
	Btm×5	18.444	24.061	23.018	30.552				
	Btm×20				3.403	5.359	8.840	1.015	
9H	Top×5	20.463	26.691	30.407	39.064				
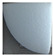	Top×10					8.921	13.308	20.193	0.8470
	Top×20	24.148	29.908	23.647	31.373	3.549	5.681	9.418	1.033
	Side×5	19.729	26.894	19.834	27.396				
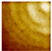	Side×20					3.477	5.454	8.982	1.010
	Btm×5	22.305	29.751	23.316	31.028				
	Btm×20					3.479	5.498	9.008	1.006
10H	Top×5	18.874	25.005	30.478	39.298				
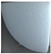	Top×10					8.576	13.155	19.795	0.8528
	Top×20	14.371	17.331	16.166	21.669	3.549	5.681	9.285	1.010
	Side×5	18.002	24.282	19.972	26.956				
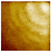	Side×20					3.549	5.811	9.231	0.978
	Btm×5	15.578	20.447	17.518	23.449				
	Btm×20					3.404	5.453	9.038	1.033
11UF	Top×5	18.715	27.603	28.171	36.272				
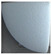	Top×10					8.577	13.153	20.437	0.9017
	Top×20	14.734	18.521	19.456	23.804	3.476	5.406	8.755	0.9764
	Side×5	15.967	22.556	18.779	25.179				
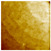	Side×20					3.407	5.450	8.837	0.996
	Btm×5	19.968	26.402	22.187	29.372				
	Btm×20					3.477	5.497	9.010	1.007
12H	Top×5	20.278	26.166	34.844	44.985				
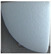	Top×10					8.806	13.456	20.290	0.8534
	Top×20	21.605	27.251	23.259	31.283	3.549	5.633	9.093	0.9841
	Side×5	17.736	23.540	20.136	26.926				
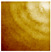	Side×20					3.479	5.546	9.008	0.997
	Btm×5	20.111	26.705	24.163	31.783				
	Btm×20					3.404	5.405	8.894	1.016
12UF	Top×5	19.912	26.831	26.279	34.215				
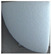	Top×10					8.345	12.768	19.338	0.8610
	Top×20	8.0216	9.704	9.718	12.498	3.331	5.171	8.282	0.9575
	Side×5	11.955	15.408	13.627	18.457				
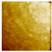	Side×20					3.478	5.590	8.953	0.979
	Btm×5	17.462	22.578	20.177	26.522				
	Btm×20					3.405	5.497	9.066	1.030
13H	Top×5	22.497	30.787	29.136	38.199				
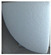	Top×10					8.694	13.230	20.244	0.8730
	Top×20	17.712	23.755	18.023	25.163	3.551	5.636	9.368	1.032
	Side×5	16.959	22.842	18.380	24.868				
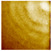	Side×20					3.548	5.634	9.231	1.009
	Btm×5	21.515	28.080	23.592	31.119				
	Btm×20					3.478	5.498	9.038	1.011
14UF	Top×5	20.203	26.333	33.779	42.504				
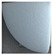	Top×10					8.577	13.228	20.194	0.8783
	Top×20	9.608	12.206	17.522	21.820	3.405	5.453	8.982	1.023
	Side×5	18.317	24.619	18.820	25.527				
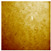	Side×20					3.407	5.452	9.035	1.032
	Btm×5	17.713	23.387	19.190	25.945				
	Btm×20					3.477	5.496	9.066	1.017
15UF	Top×5	21.939	29.007	28.892	37.296				
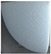	Top×10					8.461	13.001	19.898	0.8797
	Top×20	13.916	17.796	13.886	18.761	3.405	5.314	8.896	1.033
	Side×5	18.732	26.215	20.868	28.482				
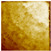	Side×20					3.477	5.546	9.032	1.002
	Btm×5	19.389	25.203	21.477	28.471				
	Btm×20					3.476	5.497	9.120	1.027
19UH	Top×5	21.186	27.276	30.910	40.059				
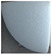	Top×10					8.921	13.890	21.203	0.8842
	Top×20	16.630	19.950	19.995	25.419	3.477	5.590	9.037	0.9946
	Side×5	17.227	23.347	19.056	25.683				
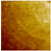	Side×20					3.478	5.545	8.923	0.982
	Btm×5	20.404	27.983	23.102	31.038				
	Btm×20					3.478	5.497	9.173	1.036
20UH	Top×5	19.233	25.623	29.022	37.581				
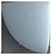	Top×10					8.922	13.670	20.916	0.8774
	Top×20	10.910	14.316	15.334	18.896	3.478	5.498	8.868	0.9804
	Side×5	16.866	22.578	19.127	25.636				
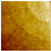	Side×20					3.479	5.591	9.149	1.014
	Btm×5	19.364	26.064	22.076	29.577				
	Btm×20					3.405	5.454	9.230	1.068
21F	Top×5	19.727	25.871	31.173	40.350				
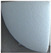	Top×10					8.577	13.153	20.045	0.8719
	Top×20	12.532	16.078	15.382	20.200	3.552	5.682	9.497	1.046
	Side×5	16.639	22.782	20.039	26.999				
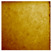	Side×20					3.403	5.406	9.893	1.201
	Btm×5	18.923	25.055	19.773	26.861				
	Btm×20					3.550	5.681	9.230	1.000
22F	Top×5	19.670	25.982	30.140	38.714				
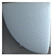	Top×10					8.576	13.077	19.898	0.8658
	Top×20	12.802	16.589	17.547	22.349	3.549	5.633	9.229	1.008
	Side×5	16.993	22.846	19.396	26.319				
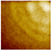	Side×20					3.477	5.497	9.009	1.006
	Btm×5	18.216	24.294	21.213	28.122				
	Btm×20					3.406	5.405	8.870	1.011

**Table 2:** Comparison of *p* values in ANOVA for original variables and factor scores. All *p* values smaller than 0.05 have been highlighted.

Obs	Variable	R-Square	*p*-Value
1	Ra (Top×5)	0.649811	0.4117
2	Rq (Top×5)	0.789014	0.1393
3	Sa (Top×5)	0.697031	0.3104
4	Sq (Top×5)	0.674443	0.3583
5	Ra (Top×20)	0.838488	0.0723
6	Rq (Top×20)	0.886009	0.0291
7	Sa (Top×20)	0.821514	0.0928
8	Sq (Top×20)	0.864371	0.0461
9	D10 (Top×10)	0.957706	0.0018
10	D50 (Top×10)	0.917584	0.0120
11	D90 (Top×10)	0.910430	0.0151
12	Span (Top×10)	0.784166	0.1469
13	D10 (Top×20)	0.820020	0.0948
14	D50 (Top×20)	0.860219	0.0499
15	D90 (Top×20)	0.786058	0.1439
16	Span (Top×20)	0.719939	0.2635
17	Ra (Side×5)	0.524485	0.6746
18	Ra (Side×5)	0.474366	0.7634
19	Rq (Side×5)	0.626568	0.4625
20	Rq (Side×5)	0.409208	0.8569
21	Sa (Side×5)	0.513080	0.6959
22	Sq (Side×5)	0.508297	0.7047
23	D10 (Side×20)	0.472391	0.7666
24	D50 (Side×20)	0.476349	0.7601
25	D90 (Side×20)	0.414649	0.8501
26	Span (Side×20)	0.524984	0.6736
27	Ra (Bottom×5)	0.815648	0.1006
28	Ra (Bottom×5)	0.824254	0.0894
29	Rq (Bottom×5)	0.885364	0.0296
30	Rq (Bottom×5)	0.833364	0.0782
31	Sa (Bottom×5)	0.584028	0.5543
32	Sq (Bottom×5)	0.626744	0.4621
33	D10 (Bottom×20)	0.741941	0.2208
34	D50 (Bottom×20)	0.558703	0.6070
35	D90 (Bottom×20)	0.743096	0.2186
36	Span (Bottom×20)	0.936378	0.0058
	Factor1	0.745209	0.2147
	Factor2	0.360598	0.9093
	Factor3	0.742239	0.2202
	Factor4	0.936331	0.0058
	Factor5	0.510720	0.7003
	Factor6	0.723604	0.2562
	Factor7	0.875494	0.0368
	Factor8	0.617470	0.4823

**Table 3:** Rotated factor pattern for Factor4 and Factor7. All Pearson correlations with an absolute value larger than 0.5 have been highlighted.

Obs	Variable	Factor4	Factor7
1	Ra (Top×5)	−0.03	0.01
2	Rq (Top×5)	−0.24	−0.09
3	Sa (Top×5)	0.26	0.20
4	Sq (Top×5)	0.24	0.19
5	Ra (Top×20)	−0.6	0.24
6	Rq (Top×20)	−0.9	0.24
7	Sa (Top×20)	0.07	0.08
8	Sq (Top×20)	0.05	0.18
9	D10 (Top×10)	0.85	0.17
10	D50 (Top×10)	0.96	0.10
11	D90 (Top×10)	0.90	−0.0
12	Span (Top×10)	0.15	−0.28
13	D10 (Top×20)	0.19	0.69
14	D50 (Top×20)	0.30	0.80
15	D90 (Top×20)	−0.02	0.80
16	Span (Top×20)	−0.37	0.47
17	Ra (Side×5)	0.03	0.12
18	Ra (Side×5)	0.16	0.12
19	Rq (Side×5)	0.12	0.09
20	Rq (Side×5)	0.01	0.13
21	Sa (Side×5)	0.12	0.16
22	Sq (Side×5)	0.10	0.21
23	D10 (Side×20)	−0.01	0.09
24	D50 (Side×20)	−0.02	0.03
25	D90 (Side×20)	−0.08	0.29
26	Span (Side×20)	−0.09	0.32
27	Ra (Bottom×5)	0.35	−0.06
28	Ra (Bottom×5)	0.07	0.18
29	Rq (Bottom×5)	0.40	−0.05
30	Rq (Bottom×5)	0.11	0.13
31	Sa (Bottom×5)	0.26	−0.05
32	Sq (Bottom×5)	0.30	−0.05
33	D10 (Bottom×20)	0.06	0.03
34	D50 (Bottom×20)	0.09	0.03
35	D90 (Bottom×20)	0.39	−0.21
36	Span (Bottom×20)	0.46	−0.033

**Table 4:** Summary of multiple comparison tests for the eight variables with a significant dependence (p<0.05) of the explanatory variables *pos*, *bus*, and *fuh* found by fitting a general linear model to the data. The “≠” symbols show the means that are significantly different.

Obs	Variable	Position (*pos*)	Build Setup (*bus*)	Full/Hollowed (*fuh*)
6	Rq (Top×20)		“D” ≠ “U”	“F” ≠ “H”
8	Sq (Top×20)		“D” ≠ “U”	
9	D10 (Top×10)	“2” ≠ “(8,1,5)”		“F” ≠ “H”
		“(7,4,3)” ≠ “5”		
10	D50 (Top×10)			“F” ≠ “H”
11	D90 (Top×10)	“4” ≠ “5”	“D” ≠ “U”	“F” ≠ “H”
14	D50 (Top×20)		“D” ≠ “U”	“F” ≠ “H”
29	Rq (Bottom×5)	“5” ≠ “7”		“F” ≠ “H”
36	Span (Bottom×20)	“3” ≠ “7”	“D” ≠ “U”	“F” ≠ “H”
	Factor4	“1” ≠ “(4,6)”	“D” ≠ “U”	“F” ≠ “H”
	Factor7		“D” ≠ “U”	

## Data Availability

The data presented in this study are openly available at https://eco3d.compute.dtu.dk/sls-roughness/ (accessed on 6 June 2023).

## Abbreviations

The following abbreviations are used in this manuscript:

ANOVA	Analysis of variance
GLM	General linear model
HSD	Honest significant difference
PA	Polyamide
Ra	Average roughness
Rq	Root mean square roughness
Sa	Arithmetical mean height
SLS	Selective laser sintering
Sq	Root mean square surface height
